# A robust zero-watermarking scheme based on non-negative matrix factorization for audio protection

**DOI:** 10.1371/journal.pone.0270579

**Published:** 2022-07-08

**Authors:** Xing Guo, Daiyu Huang, Longting Xu

**Affiliations:** College of Information Science and Technology, Donghua University, Shanghai, China; Mae Fah Luang University, THAILAND

## Abstract

The copyright problem of digital products is becoming more and more prominent. In this case, digital watermarking technology has attracted the attention of many experts and scholars in the field of information security. Among the proposed technologies, zero-watermarking technology has been favored greatly with its excellent imperceptibility. In this paper, a novel robust audio zero-watermarking scheme is designed by applying non-negative matrix decomposition algorithm to zero-watermarking technology. Firstly, the proposed scheme divides the input audio signal into fixed frames, then applies fast Fourier transform(FFT) and non-negative matrix factorization(NMF) algorithm to extract the feature vector of the original audio signal. Finally, XOR the feature vector and the digital watermark sequence to achieve the embedding of zero-watermarking. The experimental results show that the proposed scheme performs more effectively in resisting common and frame-desynchronization attacks than the existing zero-watermarking schemes.

## Introduction

Nowadays, due to the rapid development of network and computer communication technology, multimedia files can be easily distributed, shared and manipulated by people [1, 2]. Undoubtedly, this dramatically increases the demand for copyright protection. While digital watermarking is a promising technology to protect the digital audio products from tampering [3]. Specifically, zero-watermarking technology hides the copyright information in the original audio signal in an imperceptible way. It has become the main focus of researchers. However, a good zero-watermarking scheme also needs to be robust enough to resist many intentional or unintentional attacks [4, 5]. So this paper makes efforts to improve robust performance.

Watermarking technology can be divided into non-blind (including semi-blind) and blind watermarking according to whether the original audio signal and watermark are needed for watermark extraction. Non-blind watermarking requires the original audio and watermark to participate in watermark extraction, while blind watermark requires not [6]. Obviously, blind watermarking is more valuable in practical application. The audio zero-watermarking scheme designed in this paper belongs to blind watermarking. Until now, audio blind watermarking has been explored a lot.

All existed zero-watermarking schemes can be broadly categorized into two groups: time domain based and transform domain based. Methods in transform domain are more popular than in time domain due to its simplicity and effectiveness. At present, the advanced zero-watermarking schemes are mainly based on discrete cosine transform(DCT) [7], discrete wavelet transform(DWT) [8], singular value decomposition(SVD) [9] or the combination of multiple transform domain [10, 11].

The zero-watermarking schemes based on the transform domain utilize the essential characteristics of the original audio signal. Specifically, an audio zero-watermarking scheme based on dct coefficients symbol is proposed in paper [12]. It performs DCT transformation on the average value of the absolute value of each frame, then selects the maximum value of the DCT coefficient to realize the embedding of watermark. In [13], DWT transformation is executed on the host signal to get the power approximation or energy of the audio segment. Then, the watermark is extracted according to the relative energy of the continuous segment. This scheme is a suitable candidate for audio copyright protection. However, it does have weak point. Because the watermark is constructed by relationship between adjacent fragments, so this scheme is not robust against frame-desynchronization attacks. Min L et al. [11] proposes a scheme based on DCT-DWT-SVD which can effectively resist common attacks. The watermark is registered by performing SVD on the coefficients which are generated after DWT and DCT transformation. However, it still has room for further improvement. For example, when it suffers low-pass filter attack, its NC value is less than 0.96.

Furthermore, as for some state-of-the-art techniques, researchers have explored the phase spectrum of Short Time Fourier Transform (STFT) [14] to generate watermark. But it just studies three types of attacks. The various other attacks are not considered. L.Xu et al. [15] propose an audio zero-watermarking method based on sparse representation, the OMP algorithm and K-SVD algorithm are adopted. After that, authors propose a novel zero-watermarking technique based on the GFT [16]. They use the combined shift operator to construct the graph signal, and then the stable graph Fourier coefficients are selected for encoding. These two zero-watermarking schemes have good robustness against various attacks, but ours is not bad either. From the experimental results, we can find that the NC values in our scheme can reach more than 99% when resisting common attacks. So the robustness of proposed scheme and above two schemes [15, 16] is comparable.

This paper proposes an audio zero watermarking scheme based on non-negative matrix factorization so as to further improve the robust performance. The reasons why NMF algorithm used are explained below.

- Audios and images are generally represented by high-dimensional data matrix in the fields of image recognition and speech signal processing. So how to deal with multi-dimensional data has become an urgent problem for researchers. While non-negative matrix decomposition is a simple, intuitive and effective matrix decomposition technique to decompose the original high-dimensional data matrix into the basis matrix and coefficient matrix of a lower dimension [17, 18].
- More importantly, all elements in the matrix are not negative, which not only reduces the storage space, but also makes the decomposed results sparse. It is worth mentioning that this sparse feature can better represent the essential characteristics of the original audio signal [19]. Furthermore, it overcomes the limitation of SVD with non-negative restriction.
- In this paper, We use the maximum value of each group of coefficients to represent each frame of audio signal, and then encode all the maximum values. Through conducting comparative experiments with some zero-watermarking schemes, experiments results show that the proposed scheme can resist attacks better and enhance the robustness performance.

## Work contributions

Due to the rapid development of network technology, multimedia files can be easily distributed, shared and manipulated, so the demand for copyright protection is increasing day by day. A key contribution of this work is to apply NMF algorithm to zero-watermarking technology and propose a new scheme for audio copyright protection.

Audio zero-watermarking technology is a promising audio copyright protection technology with its excellent imperceptibility. Furthermore, improving its robustness is still the goal pursued by researchers. A second key contribution of this work is that we have conducted experiments on a number of known data sets and compared the results with those of three related works. Experimental results show the proposed scheme can achieve satisfactory robust performance against common attacks and frame-desynchronization attacks.

## Materials and methods

### The basic concept of non-negative matrix factorization

NMF algorithm is a matrix factorization algorithm with non-negative constraints. Given a matrix $V\in R_{+}^{n\times m}$, looking for the non-negative basis matrix $W\in R_{+}^{n\times r}$ and the non-negative coefficient matrix $H\in R_{+}^{r\times m}$ to satisfy *V* ≅ *W* × *H*. A non-negative matrix is thus decomposed into the product of two non-negative matrices. By replacing the original data matrix with the coefficient matrix, the dimensionality reduction matrix of the data feature can be obtained. Convert the matrix factorization problem into a problem of minimizing the error between two matrices. This theory can be better explained by the following formula:

$$\begin{array}{c} \text{min}\|V-V^{{}^{\prime }}\|=\sum_{ij}{\left(V_{ij}-V_{ij}^{{}^{\prime }}\right)}^{2} \end{array}$$ (1)

where *V* is the original matrix and *V*′ is the matrix to be updated (*V*′ = *W* × *H*). *V*_*ij*_ is the atom in row *i* and column *j* of the *V* matrix. In order to obtain the optimal value, the iterative formulas used in the experiment for the matrices *W* and *H* are given below.

$$\begin{array}{c} W_{ij}^{k+1}=W_{ij}^{k}\frac{{\left({\left(H^{k}\right)}^{T}*V\right)}_{ij}}{{\left({\left(H^{k}\right)}^{T}*W^{k}*H^{k}\right)}_{ij}} \end{array}$$ (2)

$$\begin{array}{c} H_{ij}^{k+1}=H_{ij}^{k}\frac{{\left({\left(W^{k}\right)}^{T}*V\right)}_{ij}}{{\left({\left(W^{k}\right)}^{T}*W^{k}*H^{k}\right)}_{ij}} \end{array}$$ (3)

The NMF algorithm is divided into two parts: training process and testing process. When training, the *W* matrix and *H* matrix are initialized randomly, where *k* represents the number of iterations. When Eq (1) converges and approaches 0 through iteration, it indicates that the input *V* matrix has been decomposed into *W* and *H* matrices. The *W* matrix will be stored. When testing, input *V* matrix and *W* matrix obtained from the training process, according to the formula in algorithm 2 to obtain the *H* matrix. Algorithm 1 and 2 describes NMF training and testing process clearly and concisely.

**Algorithm 1**: NMF-training process

Input: *V*_*n*×*m*_;

Initialize the random matrix *W*_*n*×*r*_ and matrix *H*_*r*×*m*_.

$W,H=\underset{W,H}{\text{min}}D(V||WH)$

**for** *k* ← 1 **to** *iteration number* **do**

$W_{ij}^{k+1}\leftarrow W_{ij}^{k}\frac{{\left({\left(H^{k}\right)}^{T}*\left(V\right)\right)}_{ij}}{{\left({\left(H^{k}\right)}^{T}*W^{k}*H^{k}\right)}_{ij}}$ ;

$H_{ij}^{k+1}\leftarrow H_{ij}^{k}\frac{{\left({\left(W^{k}\right)}^{T}*\left(V\right)\right)}_{ij}}{{\left({\left(W^{k}\right)}^{T}*W^{k}*H^{k}\right)}_{ij}}$ ;

*V* = *W* × *H*

**end**

Output: *W*_*n*×*r*_, *H*_*r*×*m*_

**Algorithm 2**: NMF-testing process

Input: *V*_*n*×*m*_, *W*_*n*×*r*_

**do**

$H_{ij}^{k+1}\leftarrow H_{ij}^{k}\frac{{\left({\left(W^{k}\right)}^{T}*\left(V\right)\right)}_{ij}}{{\left({\left(W^{k}\right)}^{T}*W^{k}*H^{k}\right)}_{ij}}$ ;

Output:*H*_*r*×*m*_

### The proposed zero-watermarking scheme based on non-negative matrix factorization

**Fig 1:**
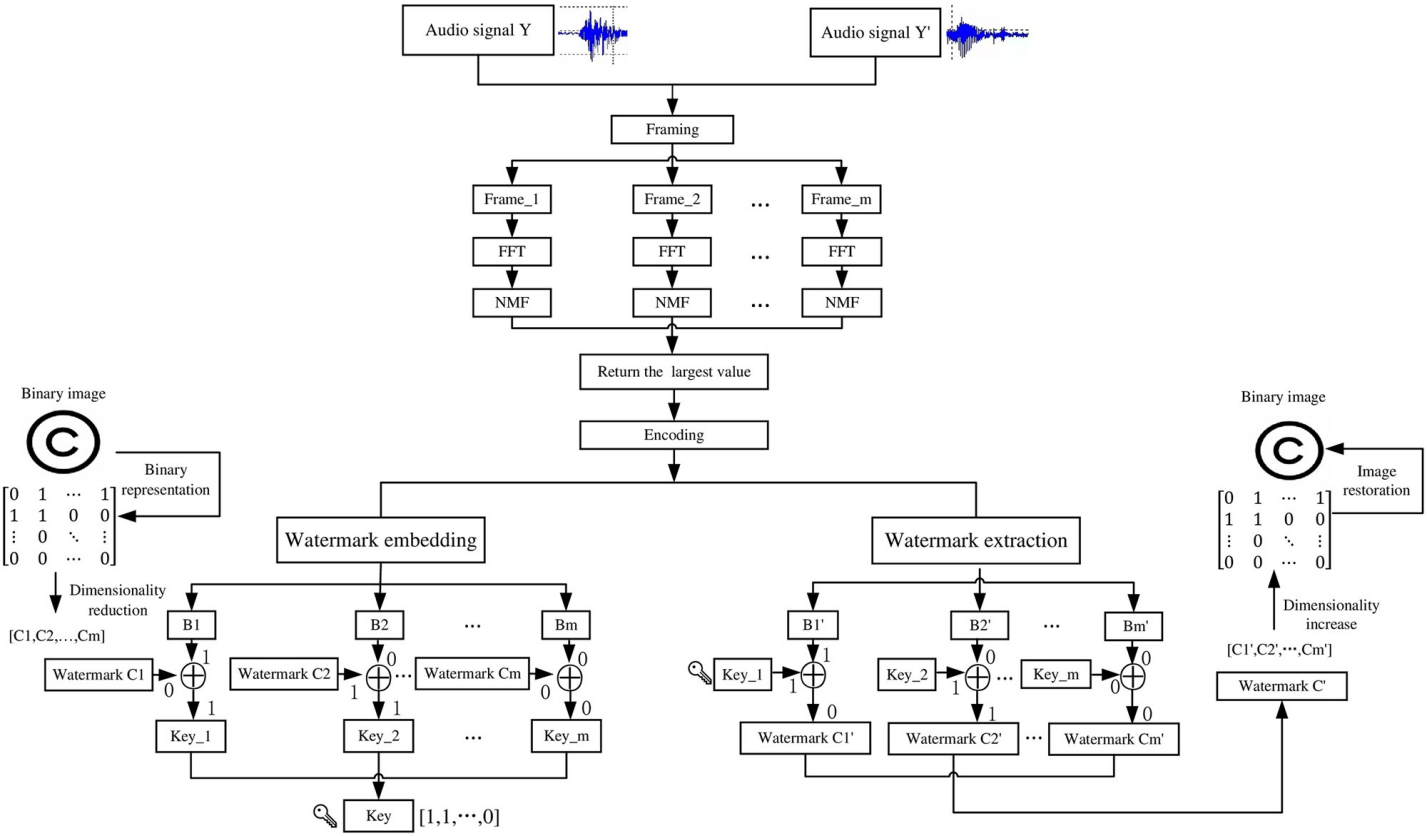
Watermark embedding and extraction process.

The scheme designed in this paper includes two modules: watermark embedding and watermark extraction. The detailed analysis is as follows. The simple and comprehensible watermark embedding and extraction process can be seen in Fig 1.

1. Watermark embedding process
  (1)Watermark image preprocessing: In this paper, the binary image *C* of *N* × *N* (*N* = 32) is used as copyright information, and the binary image is transformed into a one-dimensional signal vector by dimensionality reduction processing. Each pixel in the binary image is represented by a one-dimensional signal vector, namely:
  $$\begin{array}{c} C=\{c\left(i\right)=c\left(1\right),c\left(2\right),\cdots ,c(M),1\leq i\leq M,M=N^{2}\} \end{array}$$ (4)
  *c* represents the pixel point of the image, and *M* represents the total number of pixels of the binary image.
  (2)Audio preprocessing: At first, the number of frames of the audio signal should be consistent with the total number of pixels of the watermark image, so the input audio signal *Y* is divided into fixed *M* frames, and fast Fourier transform is performed on each frame of the signal. The length of input signal is denoted as *Y*_*len*_, and the length of each frame is denoted as *F*_*len*_.
  $$\begin{array}{c} F_{len}=floor\left(Y_{len}/M\right) \end{array}$$ (5)
  (3)Encoding: The preprocessed audio signal is decomposed by non-negative matrix to obtain the basis matrix and coefficient matrix, then take the maximum value *η*_*i*_(1 ≤ *i* ≤ *M*) in the coefficient matrix generated by each frame signal, *Mean*(*η*_*i*_) is obtained after averaging them, and a polarity vector *B*_*i*_ is formed according to the size relation between *η*_*i*_ and *Mean*(*η*_*i*_).
  $$\begin{array}{c} Mean\left(\eta_{i}\right)=\frac{\sum_{i=1}^{M}\eta_{i}}{M} \end{array}$$ (6)
  $$\begin{array}{c} B_{i}=\{\begin{array}{c} 1,\eta_{i}>mean\left(\eta_{i}\right)\\ 0,otherwise \end{array} \end{array}$$ (7)
  (4)Obtain the watermark key: XOR the polarity vector *B* and the one-dimensional watermark signal *C* to obtain the watermark key. So that we achieve the embedding of watermark.
  $$\begin{array}{c} K=B⊕C \end{array}$$ (8)
2. Watermark extraction process
  (1)Suppose the audio signal after attacks is *Y*′, then repeat step (2) of the watermark embedding above to conduct audio preprocessing.
  (2)Get the polarity vector *B*′ according to the same encoding method (3) as the watermark embedding above.
  (3)XOR the polarity vector and the key to obtain the extracted watermark signal *C*′.
  $$\begin{array}{c} C^{{}^{\prime }}=B^{{}^{\prime }}⊕K \end{array}$$ (9)
  (4)The one-dimensional watermark signal is converted to two-dimensional watermark signal, and the binary watermark image is obtained after image restoration.

## Experimental results and analyses

### Experiment preparation

The input audio signal of this experiment is from the LibriSpeech corpus [20]. It is composed of 585 hours of real voice data and corresponding text collected by 2456 speakers at a sampling rate of 24kHz. In this experiment, we set the resample point of the speech signal to 256. In order to prove the validity and the feasibility of the proposed scheme, the selected audio data set is divided into training data set and testing data set. The training data set consists of 250 sentences selected from three randomly selected people for about 20 minutes. The testing data type is divided into in-domain and out-of-domain conditions for the experiment, each testing data set contains 50 sentences and takes about 3 minutes. In-domain means that the testing data is within the range of the training data set, and out-of-domain means that the testing data is outside the range of the training data set. The detailed information is shown in Table 1. In this paper, for evaluating the robustness of the proposed scheme, we choose six different types of attacks. Below is a brief introduction to each attack.

- Noise: the SNR of the additive white Gaussian noise (AWGN) which is added to the original audio signal is 10dB, 20dB, 30dB.
- Low pass filter: a low-pass filter with cut-off frequency 5kHz is used.
- Resampling: the sampling frequency is first changed to one half of the original sampling frequency, and then changed to the original sampling frequency.
- MP3 compression: the original signal is compressed in MP3 format (128kbps).
- Re-quantization: the audio signal is quantized from 16 bits to 8 bits, and then quantized from 8 bits to 16 bits.
- Frame-desynchronization: the beginning or the end of audio signal will be cropped to 0.5 second, 1 second and 2 seconds, respectively.

**Table 1:** The Detailed experiment introduction and classification. (a,x,y and z represent a set of statements for a particular speaker).

Testing data type	Testing data	Training data set
In-domain	x	x, y, z
Out-of-domain	a	x, y, z

In this paper, the bit error rate(BER) is used to measure the difference between the extracted watermark and the original watermark, and the normalized correlation coefficient(NC) is used to measure the similarity between the extracted watermark and the original watermark, both BER and NC are the evaluation metrics to measure the robustness of the proposed scheme under various attacks. The specific calculation formulas are as follows.

$$\begin{array}{c} BER=\frac{Nb_{c}}{Nb_{o}} \end{array}$$ (10)

$$\begin{array}{c} NC\left(C,C^{{}^{\prime }}\right)=\frac{\sum_{i}^{N}C\left(i\right)C^{{}^{\prime }}\left(i\right)}{\sqrt{\sum_{i}^{N}C^{2}\left(i\right)}\sqrt{\sum_{i}^{N}{\left(C^{{}^{\prime }}\left(i\right)\right)}^{2}}} \end{array}$$ (11)

where *Nb*_*c*_ is the number of error bits, *Nb*_*o*_ means the total bits of the original watermark sequence, *C* represents the embedded watermark sequence and *C*′ represents the extracted watermark sequence, *N* is the length of watermark sequence. BER is closer to 0 and NC is closer to 1, the performance of the proposed scheme against attacks is better.

### Imperceptibility analysis

Zero-watermarking technology does not modify the original data. There is no perceptible difference between the watermarked carrier and the original carrier, so it has excellent imperceptibility.

### Robustness analysis

#### Robustness comparison between the proposed scheme and other zero-watermarking schemes under common attacks

Through BER and NC values, Table 2 compares the robust performance of the proposed scheme and some related zero-watermarking schemes [10, 12, 13] against various common attacks.

**Table 2:** Robustness comparison of the proposed scheme and other zero-watermarking schemes under common attacks, where **/** indicates average metrics BER/NC and the bold mark indicates the best number across all the schemes under each attack.

Schemes	test data	Metrics (BER/NC)						
Attacks		Noise (10dB)	Noise (20dB)	Noise (30dB)	Low pass filter	Re-sampling	MP3 compression	Re-quantization
Our	In-domain	**0.0142/0.9901**	**0.0034/0.9976**	**0.0013/0.9990**	0.0020/0.9985	0.0008/0.9994	**0.0053/0.9963**	**0.0012/0.9991**
	Out-domain	0.0151/0.9896	0.0051/0.9964	0.0015/0.9989	**0.0012/0.9991**	**0.0001/0.9999**	0.0061/0.9958	0.0014/0.9989
DCT [12]	In-domain	0.1679/0.8800	0.0874/0.9388	0.0375/0.9740	0.1088/0.9235	0.0150/0.9896	0.0425/0.9705	0.0571/0.9603
	Out-domain	0.1702/0.8783	0.1046/0.9264	0.0623/0.9566	0.1100/0.9226	0.0060/0.9958	0.0457/0.9682	0.0696/0.9514
DWT [13]	In-domain	0.1079/0.9242	0.0534/0.9628	0.0151/0.9895	0.0136/0.9906	0.0084 /0.9941	0.0175/0.9879	0.0208/0.9856
	Out-domain	0.1120/0.9208	0.0602/0.9582	0.0343/0.9762	0.0079/0.9945	0.0028/0.9980	0.0169/0.9883	0.0492/0.9658
DWT-DCT [10]	In-domain	0.1120/0.9209	0.0602/0.9567	0.0236/0.9837	0.0224/0.9845	0.0184/0.9873	0.0096/0.9933	0.0258/0.9822
	Out-domain	0.1216/0.9141	0.0621/0.8347	0.0334/0.9768	0.0170/0.9882	0.0087/0.9939	0.0136/0.9906	0.0424/0.9705

As for the proposed scheme, We observe that whether it is in-domain or out-of-domain, excluding noise attack (SNR = 10dB), the BER values under all other attacks are all below 1%, and the range of all NC values is close to or greater than 99%. This clearly illustrates the good robustness of the scheme proposed in this paper against different common attacks. For the noise attack and re-quantization attack, compared with other schemes, the proposed scheme clearly shows better BER and NC values. For the low pass filter attack, compared with the schemes [10, 12], the proposed scheme provides better BER and NC values while maintaining robustness comparable to the scheme proposed in [13]. In terms of re-sampling attack, the proposed scheme has the same robust performance as schemes [10, 12, 13], but it is also slightly better than schemes [10, 12, 13] from the values of BER and NC. The robust performance of the proposed scheme against MP3 compression attack is similar to that of scheme [10], but better than that of scheme [12, 13]. Generally speaking, the NC values of the proposed scheme are almost all around 99%, while the NC values of schemes [10, 12, 13] all range from 90% to 99%.

**Fig 2:**
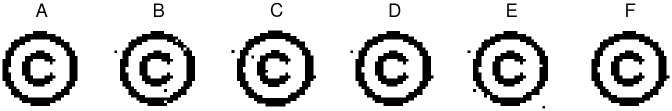
The proposed scheme used to extract the watermark images.

**Fig 3:**
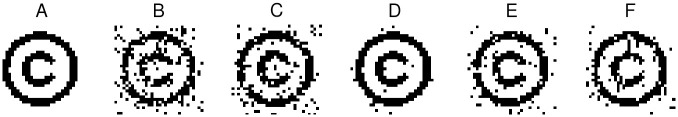
DCT scheme used to extract the watermark images.

**Fig 4:**
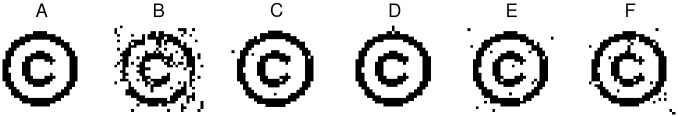
DWT scheme used to extract the watermark images.

**Fig 5:**
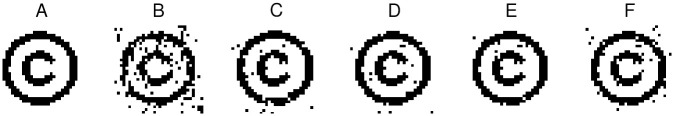
DWT-DCT scheme used to extract the watermark images.

When resisting different types of common attacks, the original watermark image and watermark images extracted by the proposed scheme and other zero-watermarking schemes are shown in Figs 2–5. A–F represents the different attack types. Specifically, A-no attack, B-noise attack, C-low pass filter attack, D-resampling attack, E-MP3 compression attack and F-re-quantization attack. We can observe that the watermark images we extracted are all clearly visible. Specifically, When resisting noise attack(10dB), the extracted watermark image of DCT [12] scheme, DWT [13] scheme and DWT-DCT [10] scheme are a bit fuzzy, showing poor robustness. This directly demonstrates better robustness of our scheme.

#### Robustness comparison between the proposed scheme and other zero-watermarking schemes under frame-desynchronization attacks

Table 3 shows BER and NC values of the proposed scheme and other zero-watermarking schemes under frame-desynchronization attacks. We can see that the proposed scheme based on blind extraction demonstrates good robustness for slight frame-desynchronization attacks, but poor robustness for serious frame-desynchronization attacks. When the duration of removed frames is less than or equal to 1 second, the BER and NC values are less than 0.3 and greater than 0.8. As the removed frames increase, the results show worse robustness.

**Table 3:** Robustness comparison of the proposed scheme and other zero-watermarking schemes under frame-desynchronization attacks.

Methods	Test Data	Metric	Frame-desynchronization attacks					
			0.5 second (begin)	1 second (begin)	2 seconds (begin)	0.5 second (end)	1 second (end)	2 seconds (end)
Our	In-domain	BER	0.1616	0.2573	0.3280	0.1073	0.1986	0.3275
		NC	0.8839	0.8107	0.7546	0.9231	0.8559	0.7544
	Out-of-domain	BER	0.1219	0.2421	0.3140	0.1731	0.2095	0.3193
		NC	0.9137	0.8227	0.7669	0.8762	0.8483	0.7620
DCT [12]	In-domain	BER	0.4956	0.5060	0.5008	0.3415	0.5046	0.5035
		NC	0.6076	0.5992	0.6042	0.7299	0.5997	0.6015
	Out-of-domain	BER	0.4906	0.4928	0.4995	0.4417	0.4945	0.4990
		NC	0.6248	0.6124	0.6047	0.6507	0.6111	0.6047
DWT [13]	In-domain	BER	0.3457	0.4158	0.4728	0.1479	0.3762	0.4600
		NC	0.7377	0.6789	0.6314	0.8885	0.7137	0.6425
	Out-of-domain	BER	0.3128	0.4392	0.4740	0.3183	0.3974	0.4778
		NC	0.7659	0.6584	0.6310	0.7591	0.6965	0.6272
DWT-DCT [10]	In-domain	BER	0.2981	0.3730	0.4668	0.1190	0.3389	0.4590
		NC	0.7757	0.7153	0.6369	0.9117	0.7437	0.6435
	Out-of-domain	BER	0.2320	0.4025	0.4586	0.2714	0.3617	0.4597
		NC	0.8309	0.6919	0.6441	0.7981	0.7253	0.6432

But on the whole, compared with other zero-watermarking schemes, the proposed scheme clearly indicates better BER and NC values. As the frame shift increases, the proposed scheme still shows better performance than other zero-watermarking schemes.

**Fig 6:**
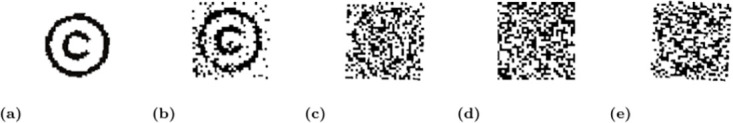
Extracted watermark images when the audio signal is clipped to 0.5 second at the beginning. (a) original watermark image; (b) image extracted by the proposed scheme; (c) image extracted by the DCT [12] scheme; (d) image extracted by the DWT [13] scheme; (e) image extracted by the DWT-DCT [10] scheme.

In addition, we select one situation where the audio signal is clipped to 0.5 second at the beginning and make a comparison with other zero-watermarking schemes. We can intuitively see from Fig 6 that When the duration of removed frames is 0.5 second, the extracted watermark image of the proposed scheme is relatively clear. While the extracted watermark images of the other three zero-watermarking schemes are very blurry. So we can conclude that the proposed scheme can resist slight frame-desynchronization attacks effectively.

#### The complexity comparison and analysis

Table 4 lists the average time cost for an audio segment and memory cost in the watermark embedding process for different schemes. As can be seen from the Table 4, compared with most schemes, the proposed scheme is more time efficient.

**Table 4:** The experimental environment and complexity comparison.

Software Hardware	MATLAB R2019a CPU Intel(R) Core(TM) i7-9700 CPU @ 3.00GHz			
schemes	Our	DCT	DWT	DWT-DCT
Time cost	0.208s	0.144s	0.225s	0.387s
Memory cost	72.0kB	-	-	-

For the proposed scheme, the core process of constructing zero-watermarking information is extracting the coefficient matrix to represent the original audio signal. Firstly, we will have a training process for input audio signal to get a base matrix which will occupy the storage space of 73,728 bytes. Then we will obtain the coefficient matrix according to Algorithm 2 which is essentially a simple matrix operation process.

## Conclusion

In this paper, a robust zero-watermarking scheme based on NMF is proposed. The audio signal is divided into fixed frames, then each frame of audio signal applies FFT and NMF to obtain the basis matrix and coefficient matrix. The maximum coefficient value of each frame signal after NMF decomposition is taken as the feature information about this frame, then the feature sequence is clustered into two classes to encode as binary sequence 0 and 1 respectively. The zero-watermarking sequence is created by performing XOR operation between the encoded sequence and the watermark sequence. Through comparative experiments, it can be observed that the proposed scheme can extract watermark image well and has good robustness under the common attacks and slight frame-desynchronization attacks. However, it still has limitations such as a need for a trusted third party. The key generated by the audio will be registered in the third party in a one to one correspondence. When extracting the watermark image, you will need to find the required key according to the mapping relationship.
